# Systematic Review and Meta-Analysis of Cognitive Behavioral Social Skills Training for Schizophrenia

**DOI:** 10.1192/j.eurpsy.2025.2145

**Published:** 2025-08-26

**Authors:** D. F. Holanda, D. V. S. Cavalcante, F. V. Zamora, L. K. C. S. Galvao, A. C. F. D. F. Santos, A. V. Zamora, N. Y. D. S. Balio, T. L. S. Barreiro, G. P. Simões

**Affiliations:** 1 Medicine, Federal University of Amazonas, Manaus; 2 Pharmacy, Federal University of Maranhao, São Luiz; 3 Medicine; 4 Federal University of Minas Gerais, Belo Horizonte; 5 Pharmacy, City University of São Paulo, São Paulo; 6 Universidade Cruzeiro do Sul, Cruzeiro do Sul; 7 Pontifícia Universidade Católica do Rio Grande do Sul, Porto Alegre; 8 Faculdades Integradas do Vale do Ivaí, Paraná, Brazil

## Abstract

**Introduction:**

Schizophrenia is a major cause of severe global functional disability with negative symptoms that greatly affect functional outcomes. These symptoms are divided into expressive (e.g., facial affect and voice tone) and experiential (e.g., amotivation and asociality) dimensions.

**Objectives:**

This study assessed the effectiveness of Cognitive Behavioral Social Skills Training (CBSST) in enhancing functioning in individuals with schizophrenia. It examined the link between defeatist performance attitudes and functional changes post-CBSST.

**Methods:**

We conducted a comprehensive search of PubMed, Embase, and Cochrane databases up to September 2024 for studies comparing CBSST with standard treatments for schizophrenia. We calculated the mean or standardized mean differences (MDs and SMD) for continuous outcomes along with 95% confidence intervals (CIs). Heterogeneity was evaluated using the I² statistics.

**Results:**

Our review included 7 studies with 462 patients, of whom 219 (47.4%) received CBSST. There were no significant differences between the groups regarding positive symptoms (SMD 0.19, 95% CI -1.01 to 0.64, I² = 95; Figure 1A), negative symptoms (SMD -0.84, 95% CI -1.85 to 0.17, I² = 93; Figure 1B), Depression Scale scores (SMD 0.18, 95% CI -0.20 to 0.57, I² = 62; Figure 1C), or the Independent Living Skills Scale (MD 0.05, 95% CI 0.04 to 0.06, I² = 0; Figure 2). However, the independent living skills scores were significantly lower in the control group.

**Image 1:**

**Figure:**
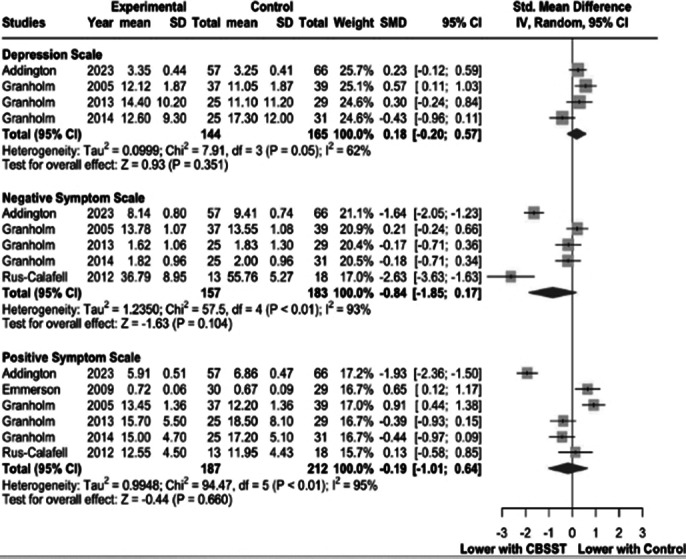

**Image 2:**

**Figure:**
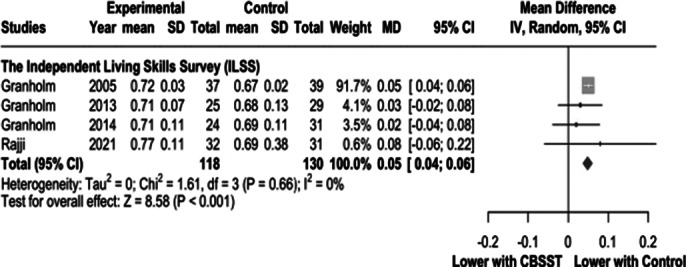

**Conclusions:**

CBSST is effective in enhancing functioning in individuals with schizophrenia. Along with other supportive goal-oriented interventions, it can alleviate symptom distress, boost motivation and self-esteem, and enhance life satisfaction. Individuals with severe defeatist performance attitudes may experience the greatest benefit from cognitive-behavioral approaches that target functional improvements.

**Disclosure of Interest:**

None Declared

